# Comparative Genomics of *Candidatus* Methylomirabilis Species and Description of *Ca*. Methylomirabilis Lanthanidiphila

**DOI:** 10.3389/fmicb.2018.01672

**Published:** 2018-07-24

**Authors:** Wouter Versantvoort, Simon Guerrero-Cruz, Daan R. Speth, Jeroen Frank, Lavinia Gambelli, Geert Cremers, Theo van Alen, Mike S. M. Jetten, Boran Kartal, Huub J. M. Op den Camp, Joachim Reimann

**Affiliations:** ^1^Department of Microbiology, Institute for Water and Wetland Research, Radboud University Nijmegen, Nijmegen, Netherlands; ^2^Department of Biotechnology, Delft University of Technology, Delft, Netherlands; ^3^Soehngen Institute of Anaerobic Microbiology, Nijmegen, Netherlands; ^4^Microbial Physiology Group, Max Planck Institute for Marine Microbiology, Bremen, Germany

**Keywords:** methylomirabilis, anaerobic methane oxidation, *NC10*, nitrite, methylotrophy

## Abstract

Methane is a potent greenhouse gas, which can be converted by microorganism at the expense of oxygen, nitrate, nitrite, metal-oxides or sulfate. The bacterium ‘*Candidatus* Methylomirabilis oxyfera,’ a member of the NC10 phylum, is capable of nitrite-dependent anaerobic methane oxidation. Prolonged enrichment of ‘*Ca.* M. oxyfera’ with cerium added as trace element and without nitrate resulted in the shift of the dominant species. Here, we present a high quality draft genome of the new species ‘*Candidatus* Methylomirabilis lanthanidiphila’ and use comparative genomics to analyze its metabolic potential in both nitrogen and carbon cycling. To distinguish between gene content specific for the ‘*Ca*. Methylomirabilis’ genus and the NC10 phylum, the genome of a distantly related NC10 phylum member, CSP1-5, an aerobic methylotroph, is included in the analysis. All genes for the conversion of nitrite to N_2_ identified in ‘*Ca.* M. oxyfera’ are conserved in ‘*Ca.* M. lanthanidiphila,’ including the two putative genes for NO dismutase. In addition both species have several heme-copper oxidases potentially involved in NO and O_2_ respiration. For the oxidation of methane ‘*Ca.* Methylomirabilis’ species encode a membrane bound methane monooxygenase. CSP1-5 can act as a methylotroph, but lacks the ability to activate methane. In contrast to ‘*Ca.* M. oxyfera,’ which harbors three methanol dehydrogenases (MDH), both CSP1-5 and ‘*Ca.* M. lanthanidiphila’ only encode a lanthanide-dependent XoxF-type MDH, once more underlining the importance of rare earth elements for methylotrophic bacteria. The pathways for the subsequent oxidation of formaldehyde to carbon dioxide and for the Calvin–Benson–Bassham cycle are conserved in all species. Furthermore, CSP1-5 can only interconvert nitrate and nitrite, but lacks subsequent nitrite or NO reductases. Thus, it appears that although the conversion of methanol to carbon dioxide is present in several NC10 phylum bacteria, the coupling of nitrite reduction to the oxidation of methane is a trait so far unique to the genus ‘*Ca.* Methylomirabilis.’

## Introduction

Methane (CH_4_) is an important fuel for households and industry, but also a potent greenhouse gas (Forster et al., 2007) with radiative forcing 25 to 30 times higher than carbon dioxide (CO_2_) on a 100-year scale (Denman et al., 2007). As a consequence of anthropogenic activity, the concentration of methane in the atmosphere has risen exponentially over the past 200 years (Myhre et al., 2013; IPCC, 2014). Global warming is a worldwide concern and deepening our understanding of the sources and sinks of methane is essential to improve climate predictions and devise mitigation strategies.

Approximately 20–30% of methane is produced via thermal decomposition of organic matter within the Earth’s crust, whereas the remaining 70–80% is biogenic and originates from natural ecosystems and anthropogenic activities (Conrad, 2009). This biogenic methane is mainly produced by methanogenic archaea during the decomposition of organic matter in anaerobic habitats (Thauer and Shima, 2008) and by aerobic marine microorganisms in the ocean that can cleave methylphosphonate (Karl et al., 2008; Metcalf et al., 2012). Before methane reaches the atmosphere, most of it (50–80%) is oxidized by methanotrophs that use methane as electron donor and oxygen, nitrate, nitrite, metal-oxides or sulfate as electron acceptors (Raghoebarsing et al., 2006; Conrad, 2009; Knittel and Boetius, 2009; Haroon et al., 2013; Ettwig et al., 2016; in ‘t Zandt et al., 2018). Therefore, these microorganisms play key roles in the major global elements cycles.

Aerobic methanotrophs belonging to the *Alpha-* and, *Gamma-proteobacteria* and the *Verrucomicrobia* (Hanson and Hanson, 1996; Op den Camp et al., 2009) have been widely investigated. The first indications of methane oxidation in the absence of oxygen came from studies on sulfate-rich deep-sea sediments containing methane hydrates (Barnes and Goldberg, 1976; Whalen and Reeburgh, 1996). It was later shown that sulfate-dependent methane oxidation is conducted by consortia of methanotrophic archaea and sulfate-reducing *Delta-proteobacteria*, and by methanotrophic archaea on their own (Boetius et al., 2000; Knittel and Boetius, 2009; Milucka et al., 2012; Scheller et al., 2016). Nitrate and metal-oxide dependent methane oxidation can be conducted by ANME-2 methanotrophic archaea (Haroon et al., 2013; Ettwig et al., 2016; Cai et al., 2018; for recent reviews, see Welte et al., 2016; Timmers et al., 2017). All methane-oxidizing archaea use the reverse methanogenesis pathway to convert CH_4_ to CO_2_.

**FIGURE 1:**
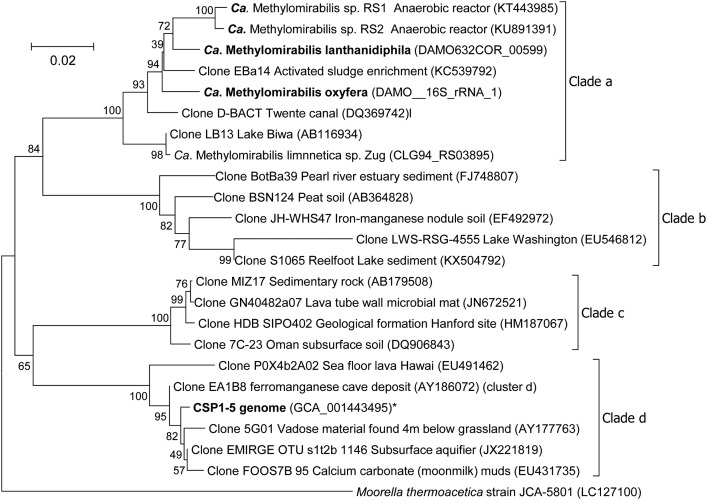
Phylogenetic tree of the 16S rRNA gene sequences of representatives of the NC10 phylum. The evolutionary history was inferred using the Neighbor-Joining method. The optimal tree with the sum of branch length = 0.52876954 is shown. The percentage of replicate trees in which the associated taxa clustered together in the bootstrap test (500 replicates) are shown next to the branches. The tree is drawn to scale, with branch lengths in the same units as those of the evolutionary distances used to infer the phylogenetic tree. The evolutionary distances were computed using the Jukes-Cantor method and are in the units of the number of base substitutions per site. The analysis involved 24 nucleotide sequences. All ambiguous positions were removed for each sequence pair. There were a total of 1573 positions in the final dataset. *Moorella thermoacetica* was used as outgroup. ^∗^The 16S rRNA gene of CSP1-5 is present on contig LDXR01000002 at position 245454-247004.

The only known organism that can couple methane oxidation to the reduction of nitrite to N_2_ is ‘*Candidatus* Methylomirabilis oxyfera’ (Ettwig et al., 2010), the first cultured member of the NC10 phylum. Using molecular techniques representatives of the NC10 phylum were detected in diverse environments ranging from peatlands (Zhu et al., 2012), paddy soils (He et al., 2016) and wastewater treatment plants (Luesken et al., 2011) to marine environments (He et al., 2015) and deep stratified lakes (Graf et al., 2018). Phylogenetic analysis divided the 16S rRNA gene sequences obtained into four different clades with bona fide methane-oxidizing ‘*Ca.* M. oxyfera’ clustering in clade A (Ettwig et al., 2009) (**Figure 1**). In contrast to the methane-oxidizing archaea, ‘*Ca.* M. oxyfera’ uses the canonical aerobic methane oxidation pathway and employs all essential protein complexes found in aerobic methanotrophs. The oxygen needed for methane activation is hypothesized to be generated via a unique pathway (Ettwig et al., 2010). This microorganism first reduces nitrite to nitric oxide (NO), followed by the disproportionation of two molecules of NO to O_2_ and N_2_ (Ettwig et al., 2010, 2012). Subsequently, the produced O_2_ is used for the activation of methane into methanol by methane monooxygenase. The second step in the canonical pathway of aerobic methane oxidation is the conversion of methanol to formaldehyde, catalyzed by either a calcium-dependent MxaFI-type or a lanthanide-dependent XoxF-type methanol dehydrogenase (MDH). Recently it was discovered that lanthanides are very important for expressing functional XoxF-type MDH (Pol et al., 2014; Vu et al., 2016). As the genome of ‘*Ca.* M. oxyfera’ contains both MxaFI and XoxF type MDH genes, an NC10 enrichment culture was supplemented with the lanthanide cerium to release a potential growth limitation. In addition, nitrate was omitted from the medium to decrease the abundance of the nitrate-dependent ‘*Ca*. Methanoperedens sp.,’ in the culture.

After 2 years of operation in the presence of cerium and absence of nitrate, the metagenome of the enrichment culture was determined. Analysis of the 16S rRNA gene after assembly revealed that the community had shifted toward a new ‘Ca. Methylomirabilis’ species as the dominant organism. Here, we describe the high quality draft genome of this new NC10 bacterium from the ‘Ca. Methylomirabilis’ genus, capable of performing nitrite-dependent anaerobic methane oxidation. Genome comparisons with a putative aerobic methanol-oxidizing NC10 bacterium (CSP1-5) described recently (Hug et al., 2016), and the original ‘Ca. Methylomirabilis’ genome (Ettwig et al., 2010) were made. CSP1-5 clusters within clade D of the NC10 phylum (**Figure 1**) and is used in this analysis as an outlier to delineate the genomic traits at a phylum and genus level.

## Materials and Methods

### Enrichment Culture

The enrichment culture was operated as a continuous sequencing batch reactor (Applikon Biotechnology B.V., nominal volume 6 L). The seed inoculum was obtained from an enrichment culture originally inoculated with ditch sediment from Ooijpolder (Ettwig et al., 2009, 2010). The enrichment culture and the medium were kept anoxic by continuous sparging with a gas mixture of methane and carbon dioxide (95:5 volume ratio) and a mixture of argon and carbon dioxide (95:5 volume ratio), respectively. The pH was maintained at 7.3 ± 0.1 (using KHCO_3_), temperature was kept at 30°C. The volume of the enrichment was kept constant through a level sensor-controlled pump in sequential feed and rest cycles and stirred at a constant speed of 100 rpm during feed periods. The hydraulic retention time was 4 days.

The final composition of the medium supplied was: MgSO_4_ 0.78 mM, CaCl_2_ 1.96 mM, KH_2_PO_4_ 0.73 mM. The nitrite concentration of <100 μM was maintained in the reactor by adjusting the nitrite concentration in the influent medium, which ranged from 1 to 40 mM according to consumption rate of the enrichment culture. Nitrite and nitrate concentrations were determined by colorimetric strip measurements (Merck).

The composition and concentrations of trace elements in the medium was adapted from the original trace element solution used in Ettwig et al. (2010). Based on the discovery of the importance of lanthanides for methanotrophic bacteria (Pol et al., 2014) we included cerium as a trace element. The final trace element concentration in the medium was: ZnSO_4_ 0.26 μM, CoCl_2_ 0.15 μM, CuSO_4_ 2.82 μM, NiCl_2_ 0.24 μM, H_3_BO_3_ 0.07 μM, MnCl_2_ 0.30 μM, Na_2_WO_4_ 0.05 μM, Na_2_MoO_4_ 0.12 μM, SeO_2_ 0.14 μM, CeCl_2_ 0.12 μM, and FeSO_4_ 5.4 μM.

### Trace Element Analysis

Concentrations of trace elements were determined by inductively coupled plasma-mass spectrometry (ICP-MS) with a quadrupole Xseries I spectrometer (Thermo Fisher Scientific), in a matrix of 1% HNO_3_. All standards were certified solutions: 109498 (multi element standard Merck), 111355 (certiPUR 23 element standard Merck), Tungsten single element standard (BDH^®^ ARISTAR^®^), 119796 (Selenium standard solution Merck), 70227 (Molybdenum standard solution Merck) and 70311 (Cerium standard solution Merck).

### DNA Extraction, Library Preparation, and Sequencing

DNA was extracted from the enrichment culture using an organic extraction (CTAB) protocol, the FastDNA SPIN kit (MP Biomedicals) and the Powersoil kit (MoBio Laboratories Inc.) as described previously (Speth et al., 2016). Approximately 100 ng of total DNA obtained for the three DNA extractions was used to generate a library for Ion Torrent sequencing, using the Ion Xpress^TM^ Plus Fragment Library Kit (Thermo Fisher Scientific, Waltham, MA, United States). Shearing was performed using the Bioruptor (Diagenode, Seraing, Belgium) for six cycles (1 min on, 1 min off). Size selection for a 400 bp library was performed using a 2% E-Gel^®^SizeSelect^TM^ Agarose gel (Invitrogen, United States). The library was amplified for eight cycles. DNA concentration and size was measured using a Bioanalyser^TM^ instrument and the Agilent High Sensitivity DNA kit (Agilent Technologies, Santa Clara, CA, United States) After dilution to a final concentration of 26 pM, fragments were amplified to Ion Sphere particles using the Ion One Touch^TM^ 2 Instrument and Ion PGM^TM^ Template OT2 400 Kit (Thermo Fisher Scientific, Waltham, MA, United States) according to the manufacturer’s instructions. After enrichment of the Template-Positive Ion Sphere Particles using the Ion One Touch^TM^ ES, sequencing was done using the Ion PGM 400 Sequencing Kit according to the manufacturers’ instructions (Thermo Fisher Scientific, Waltham, MA, United States).

### Genome Assembly, Binning, and Error Correction

The obtained sequencing reads were trimmed to remove low quality base-calls (end trimming with a quality limit of 0.05 and discarding reads < 50 bp), yielding 6,495,404 trimmed reads. The reads obtained from the three samples were co-assembled *de novo* (word size 35, bubble size 5000, minimum size 1000 bp) using the CLC genomics workbench (v8.03, CLCbio). The assembly generated 14,373 contigs, ranging from 1,000 bp to 228,265 bp in length with an N50 of 2,403 bp. The draft genome of the new ‘*Ca.* Methylomirabilis’ species was manually binned from the metagenome based on clustering of coverage depth and GC content. Metagenome binning was performed using R^1^ and custom scripts available at www.github.com/dspeth. Errors characteristic of Ion Torrent sequencing, such as homopolymers (Bragg et al., 2013) were corrected manually using the CLC genomics workbench and scripts available at www.github.com/dspeth. Annotation was done with PROKKA (v1.10) (Seemann, 2014), using the previously published genome (Ettwig et al., 2010) as a primary annotation source. The quality of the genome was estimated using a single-copy marker gene analysis with CheckM (Parks et al., 2015).

### Comparative Genome Analysis

Protein BLAST (BLAST+ suite version 2.3.1) (Altschul et al., 1990) using default parameters was used to assess the conservation of proteins in the two ‘*Ca.* Methylomirabilis’ species and the NC10 genome CSP1-5. The BLASTp analysis resulted in paired comparisons of annotated genes and we focused on the proteins belonging to the core metabolic pathways (**Table 2**) and described their degree of conservation based on global amino acid identity and conserved active site residues.

### Fluorescence *in Situ* Hybridization

The medium fed to the stable enrichment culture was changed in 2014 by removing nitrate and increasing trace element concentrations (see above). In 2016, after approximately 2 years since the change in medium composition, a biomass sample of 1.5 mL was pelleted for 3 min at 4000 G. The pellet was then washed twice with 1 ml phosphate-buffered saline (PBS: 130 mM NaCl and 10 mM phosphate buffer pH 7.4). After two washing steps, the sample was pelleted, re-suspended in 300 μL of PBS and mixed with 900 μL 4% paraformaldehyde for fixation (1:3 ratio). The fixation sample was kept mixing overnight at 10 RPM at a temperature of 4°C. Probes used were S-^∗^-DBACT-1027-a-A-18 for ‘*Ca*. Methylomirabilis’ spp. and S-^∗^-AAA-FW-641 for ‘*Ca*. Methanoperedens’ spp. (Ettwig et al., 2016). Hybridization was performed as described previously (Ettwig et al., 2009) using 40% formamide. Images were captured with a Zeiss Axioplan 2 microscope equipped with a CCD camera, and processed with the Axiovision software package (Zeiss, Germany).

### Phylogenetic Analysis

All phylogenetic analyses for 16S rRNA genes and functional genes included in this work were performed using MEGA 6.0. Alignments of protein sequences were done with the MUSCLE algorithm (Edgar, 2004) and imported into the MEGA6 software and manually inspected and corrected (Tamura et al., 2013). Phylogenetic distance trees were calculated using different methods. The confidence of the phylogenetic tree was assessed by bootstrap analysis using 1,000 replicates.

The phylogenetic composition of the microbial community was examined by mapping all trimmed reads against the SILVA rRNA reference alignment^2^ (SSU NR 99 release 128, Quast et al., 2012). The 16S sequences were uploaded to the SILVAngs pipeline to obtain a description of the microbial composition at different taxonomic levels (Yilmaz et al., 2014).

## Results and Discussion

A continuous enrichment culture performing anaerobic methane oxidation coupled to nitrite reduction (Ettwig et al., 2009) was the start of this study. During six additional years of continuous operation, the enrichment culture maintained an average nitrite conversion rate of 6.3 μmol mg protein^-1^ day^-1^, which is close to the consumption rate observed for the original enrichment described by Ettwig et al. (2009) (7.0 μmol mg protein^-1^ day^-1^).

The original enrichment was maintained with nitrate (1.5 mM, approximately 5–10% of influent nitrite concentration) in the medium, to prevent possible redox imbalance due to nitrite limitation. In 2014, the medium was revised and did no longer include nitrate in order to reduce the relative abundance of the archaeal methane oxidizers that rely on nitrate as electron acceptor. Excluding nitrate from the reactor system reduced the archaeal population from 10 to 15% to below the detection threshold of fluorescence *in situ* hybridization (FISH) analysis (data not shown). In addition to omitting nitrate, the medium was supplemented with cerium (0.12 μM CeCl_2_) to support the expression of active lanthanide-dependent MDHs (Pol et al., 2014), which were shown to be present in the genome of ‘*Ca.* M. oxyfera’ (Ettwig et al., 2010; Wu et al., 2015). In addition to the decrease in archaea, these changes resulted in a shift of the dominant ‘*Ca.* Methylomirabilis’ in the enrichment culture within 2 years after modifying the growth medium.

### Microbial Community Analysis

**FIGURE 2:**
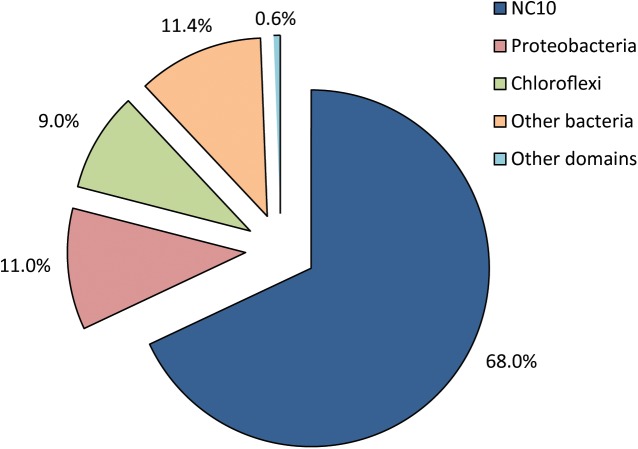
Relative distribution and classification of all 16S rRNA reads from the metagenome of a nitrite-dependent methane oxidizing enrichment culture. 2,416 sequences were successfully classified into 746 OTUs (operational taxonomical units) using the SILVAngs pipeline v128.

DNA was extracted and sequenced yielding a high coverage metagenome of the enrichment culture. All reads matching the 16S rRNA gene were extracted and analyzed in the SILVAngs pipeline 1.3. Out of 2,489 total sequences, 2,416 were successfully classified into 746 OTUs (operational taxonomic units), 99% of which were bacterial (**Figure 2**). Out of the 2,398 bacterial 16S rRNA gene reads, 1,613 sequences (67.3%) mapped to the NC10 phylum, all belonging to genus ‘*Ca.* Methylomirabilis.’ This reads could be assembled to a unique 16S rRNA gene different from known ‘*Ca*. Methylomirabilis’ spp. (see also below). Proteobacteria and Chloroflexi constituted 10.8% and 8.4% of bacterial 16S rRNA gene reads, respectively. The remaining 13.5% of bacterial 16S rRNA gene reads belonged to 20 different phyla. In accordance with the absence of general archaeal and specific ‘*Ca.* Methanoperedens’ signals in FISH analysis (data not shown), only a single 16S rRNA read was classified as archaeal, and mapped to the genus ‘*Ca* Methanoperedens.’

### Genome Analysis and Comparison

From the metagenome, a high-quality draft genome belonging to the ‘*Ca.* Methylomirabilis’ genus was obtained (**Table 1**). This genome consisted of 90 contigs with an estimated completeness of 95.4% and contamination of less than 4.3%. The 16S rRNA gene sequence present in the assembled genome showed 97.5% identity to both ‘*Ca.* M. oxyfera’ (Ettwig et al., 2010) and ‘*Ca.* M. sinica’ (He et al., 2016), and 96.3% to ‘*Ca.* M. limnetica’ (Graf et al., 2018), and clustered within clade A of the NC10 phylum (**Figure 1**). Based on 16S rRNA gene identity and the average nucleotide identity (ANI = 82.3%) of the new genome compared to the ‘*Ca.* M. oxyfera’ genome (Ettwig et al., 2010) the enriched species can be considered as novel member of the ‘*Ca.* Methylomirabilis’ genus. For this organism we propose the name ‘*Ca.* Methylomirabilis lanthanidiphila’ (*lanthanidum* lanthanide *phile* having an affinity for).

**Table 1:** General properties of the three NC10 phylum genomes.

	Genome size (Mb)	Number of contigs	GC content (%)	Completeness (%)	Contamination (%)
‘*Ca*. M. oxyfera’	2.75	1	58.58	96.26	2.59
‘*Ca.* M. lanthanidiphila’	3.05	90	59.87	95.44	4.27
CSP1-5	2.8	40	62.39	97.41	8.05


‘*Ca.* M. lanthanidiphila’ could have been present in the initial enrichment cultures, but most likely only in very small amounts. The addition of cerium in the growth medium is most likely the controlling factor for its appearance. Alternatively, Gambelli et al. (2016) described a bacteriophage infection of ‘*Ca.* Methylomirabilis sp.’ cultivated in a different bioreactor system, which was speculated to contribute to shaping the microbial community without affecting nitrite-dependent methane oxidation rates of the reactor. However, a viral infection was not observed in this reactor system and thus seems to be an unlikely cause for the present dominance of the novel ‘*Ca.* Methylomirabilis’ species.

To establish the metabolic potential of ‘*Ca.* M. lanthanidiphila’ and to determine the degree of amino acid identity of key catabolic enzymes to ‘*Ca.* M. oxyfera’ we performed a comparative genome analysis (**Table 2**). In this analysis the genome of CSP1-5, a representative of clade D within the NC10 phylum was also included. The CSP1-5 genome was recently described suggesting that this microorganism could be a potential aerobic methylotroph incapable of oxidizing methane (Hug et al., 2016).

**Table 2:** Proteins potentially involved in the dissimilatory nitrogen and carbon reactions identified in the three NC10 phylum genomes.

Reaction	‘*Ca.* M. oxyfera’	‘*Ca.* M. lanthanidiphila’	CSP1-5
Nitrate reduction	Nar/Nxr(1)^∗^, Nap(1)	Nar/Nxr(2)^∗^, Nap(1)	Nar(2)^∗^
Nitrite reduction	NirS(1)	NirS(1)	–
Nitric oxide dismutation	NOD(2)	NOD(2)	–
Nitric oxide reduction	qNor(1), sNor(1), gNor(1)	qNor(1), sNor(1), gNor(1)	–
Oxygen reduction	Cco(1)	Cco(1)	Cco(2)
Nitrous oxide reduction	–	–	–
Hydroxylamine oxidation	Hao(1)	Hao(1)	–
Methane oxidation	pMMO(1)	pMMO(1)	–
Methanol oxidation	Mxa(1), Xox(2)	Xox(1)	Xox(1)
Formaldehyde oxidation	H_4_F(1), H_4_MPT(1)	H_4_F(1), H_4_MPT(1)	H_4_F(1), H_4_MPT(1)
Formate oxidation	Fdh(3)	Fdh(2)	Fdh(2)
Carbon fixation	CBB(1)	CBB(1)	CBB(1)


*The number of proteins found in the specific species is indicated between parentheses. ^∗^Nar/Nxr can either perform nitrate reduction or nitrite oxidation.*

### Conversion of Nitrogenous Compounds

Although oxidation of methane coupled to nitrate reduction was not observed for ‘*Ca.* M. oxyfera’ (Ettwig et al., 2008), two gene clusters encoding both the membrane-bound (NarGHI; Damo_0774-0779) and periplasmic (NapAB; Damo_2410-11) nitrate reductases were encoded in its genome. NapAB was also detected in the proteome while the *nar* gene products were not (Ettwig et al., 2010). Both gene clusters were also present in the ‘*Ca.* M. lanthanidiphila’ genome (NarGHI: mela_02380-02385; 70–92% identity, NapAB: mela_582-583; 77 and 89% identity) and surprisingly an additional copy of the *nar* operon was also found (mela_00628-00631; 24–31% identity). Two *nar* operons were present in the CSP1-5 genome (XU15_C0015C00G0010-13 and XU15_C0015G0014-18), but NapAB was not detected. Conversely, the recently described ‘*Ca.* M. limnetica’ (Graf et al., 2018) encodes NapAB, but lacks a *nar* operon.

While the canonical Nar-type nitrate reductases (NarGHI) are involved in dissimilatory nitrate reduction, the related nitrite oxidoreductases (NxrABC) from aerobic nitrite-oxidizing bacteria and anaerobic ammonium-oxidizing bacteria (anammox) perform nitrite oxidation to nitrate. Phylogenetic analysis of the catalytic NarG/NxrA subunits present in ‘*Ca.* M. oxyfera,’ ‘*Ca.* M. lanthanidiphila’ and CSP1-5 revealed an interesting result (Supplementary Figure S1). The catalytic subunits of the two highly similar membrane-bound nitrate reductases identified in the ‘*Ca.* M. oxyfera’ genome (Damo_0778) and the new ‘*Ca.* M. lanthanidiphila’ genome (mela_02381) cluster with NxrA subunits from *Nitrobacter/Nitrococcus* species and might thus be able to catalyze nitrite oxidation. The additional NarG found in ‘*Ca.* M. lanthanidiphila’ (mela_00628) and one of the two present in CSP1-5 (XU15_C0015C00G0013) grouped with NarG subunits of denitrifiers, indicating that these proteins could be involved in nitrate reduction to nitrite. The NarG/NxrA subunit of the second Nar system identified in CSP1-5 (XU15_C0015C00G0018) clusters with NxrA from nitrite-oxidizing *Nitrospina*, *Nitrospira* and anammox species and its likely function is the oxidation of nitrite to nitrate. However, there is no experimental evidence to support the function of these enzymes neither as nitrate reductase nor as nitrite oxidoreductase, and thus future studies should address their respective metabolic roles.

The gene encoding the periplasmic nitrate reductase NapAB was conserved between both ‘*Ca.* Methylomirabilis’ species and was part of a larger gene cluster in which the cytochrome *cd_*1*_* nitrite reductase (NirS), which reduces nitrite to NO, and the genes required for heme *d* biosynthesis were encoded (Damo_2408-2415; mela_00580-586). The genes encoding NirS in both ‘*Ca*. Methylomirabilis’ species were highly similar to each other (97%). The CSP1-5 genome lacked the *nap*AB and *nir*S, but encoded for a multi-copper oxidase previously described as *nir*K-type nitrite reductase (Hug et al., 2016; XU15_C0012C00G0090). However, BlastP analysis showed that this gene encodes a protein more likely related to laccase-like multi-copper oxidases known to catalyze the oxygen-dependent degradation of a variety of organic substrates. In addition, a conserved aspartate residue essential for nitrite reduction was absent in the amino acid sequence of this protein (Boulanger et al., 2000). No other genes involved in nitrite reduction were identified in CSP1-5, limiting its involvement in nitrogen cycling to the interconversion of nitrate and nitrite.

In ‘*Ca.* M. oxyfera’ two molecules of NO, formed by the NirS nitrite reductase, are proposed to be dismutated into N_2_ and O_2_ by a putative NO dismutase (NOD) (Ettwig et al., 2010). Two candidates for this novel enzyme were identified in the ‘*Ca.* M. oxyfera’ genome, NOD1 (Damo_02434) and NOD2 (Damo_02437). These were homologous to the quinol-dependent NO reductases (qNOR), but displaying amino acid changes at positions proposed to be essential for NO disproportionation (Ettwig et al., 2012; Reimann et al., 2015). Similar to ‘*Ca.* M. limnetica’ both putative NODs were conserved in ‘*Ca.* M. lanthanidiphila’ (mela_02433 and mela_02434; 95% and 96% identity, respectively) including all the relevant amino acid changes. The conservation of these enzymes underlines their importance in the metabolism. However, the characterization of a NOD enzyme remains to be achieved to prove experimentally that nitric oxide disproportionation is indeed catalyzed by this enzyme.

Besides the two putative NODs, three NO reductases were encoded in the genomes of both ‘*Ca.* M. oxyfera’ and ‘*Ca.* M. lanthanidiphila,’ a canonical qNOR (Damo_1889, mela_0936; 89% identity), a gNOR (Damo_1118-1119, mela_2627-2626; 90% and 76% identity) and a sNOR (Damo_0801-0802, mela_2378-2377; 87% and 82% identity) (Hemp and Gennis, 2008; Reimann et al., 2015). ‘*Ca*. M. limnetica’ only possesses the qNOR. Because the product of all three NO reductases, N_2_O, was only measured in low amounts (Ettwig et al., 2010) and no N_2_O reductase could be identified in the genome of either ‘*Ca.* Methylomirabilis’ species, it remains to be shown what the physiological roles of these three enzymes are. They might enable the organism to quickly respond to nitrosative stress and prevent the build-up of toxic NO concentrations (Reimann et al., 2015).

Aside from NO production from nitrite by NirS, an additional source of NO in ‘*Ca*. M. oxyfera’ is hydroxylamine oxidation. Already at micromolar concentrations ammonia is oxidized by methane monooxygenases (MMOs) as a side reaction, producing hydroxylamine (Nyerges and Stein, 2009; Stein and Klotz, 2011; Stein et al., 2012; Mohammadi et al., 2017). While it is unknown whether methanotrophs are able to conserve energy for growth from the oxidation of hydroxylamine, several do harbor the gene for hydroxylamine oxidoreductase (HAO) (Bergmann et al., 2005; Poret-Peterson et al., 2008; Campbell et al., 2011; Mohammadi et al., 2017). HAOs perform the three electron oxidation of hydroxylamine to nitric oxide in both aerobic and anaerobic ammonia oxidizers (Hooper and Terry, 1979; Kostera et al., 2010; Maalcke et al., 2014; Caranto and Lancaster, 2017). Both ‘*Ca.* M. oxyfera’ and ‘*Ca.* M. lanthanidiphila’ encoded for an HAO-like protein (Damo_2473 and mela_03059; 88% identity), and were thus equipped to oxidize hydroxylamine. Strikingly the HAO-like protein was absent in the ‘*Ca*. M. limnetica’ metagenome bin (Graf et al., 2018). In line with the absence of methane monooxygenase in CSP1-5 no HAO homolog was found in its genome.

Although the two cultured ‘*Ca.* Methylomirabilis’ species are obligate anaerobes both genomes encode for the mitochondrial-like *aa*_3_-type cytochrome *c* oxidase (Damo_1162-1666, mela_00197-200; 80–90% identity), which could potentially respire part of the oxygen produced by NO dismutation (Wu et al., 2011). Since CSP1-5 is an aerobic organism, it encodes for an A1- (XU15_C0015G0022-23) and an A2- (XU15_C0015G0039-42) type cytochrome *c* oxidase.

### Methane and Methanol Conversion

In ‘*Ca.* M. oxyfera’ methane is converted to methanol by a particulate methane monooxygenase (pMMO). Sequence comparison of the *pmo*A (Damo_2450), *pmo*B (Damo_2448) and *pmo*C (Damo_2451) genes, encoding the different subunits of this enzyme with other known *pmo* genes, showed that the ‘*Ca.* M. oxyfera’ genes form a distinct phylogenetic group (Ettwig et al., 2010). Nevertheless, all secondary structure elements and amino acids involved in metal binding are conserved in *pmo*A, *pmo*B and *pmo*C of ‘*Ca.* M. oxyfera’ (Reimann et al., 2015). The ‘*Ca.* M. lanthanidiphila’ and ‘*Ca.* M. limnetica’ genomes also encoded for one pMMO (pmoA, mela_02442, pmoB, mela_02441, pmoC, mela_03065) and lacked genes encoding soluble methane monooxygenase (sMMO). All three pMMO subunits had high sequence identity at the amino acid level to the *pmo* genes of ‘*Ca.* M. oxyfera’ (*pmo*A, 94% identity; *pmo*B, 95% identity; *pmo*C, 92% identity). The CSP1-5 genome lacked all genes required for the conversion of methane to methanol by either sMMO or pMMO (Hug et al., 2016).

In ‘*Ca.* M. oxyfera’ methanol is oxidized through a periplasmic pyrroloquinoline quinone (PQQ) containing MDH (Wu et al., 2015). The genome of ‘*Ca.* M. oxyfera’ harbors three different MDHs in one large gene cluster, but lacks the genes required for PQQ biosynthesis (Wu et al., 2015). Damo_0112-122 encodes for the canonical MxaFI MDH with its accessory proteins. This enzyme carries calcium as an essential cofactor, which together with PQQ forms the active site. The other two MDH enzymes are XoxF-type, and belong to the XoxF1 (Damo_0124) and XoxF2 (Damo_0134) clusters, respectively (Keltjens et al., 2014). XoxF-type MDHs were recently shown to bind lanthanides as a metal cofactor instead of calcium (Keltjens et al., 2014; Pol et al., 2014). Strikingly, the MDH from ‘*Ca*. M. oxyfera’ was purified as a heterodimer consisting of the XoxF1 large (Damo_0124) and the MxaI small subunit (Damo_0115) (Wu et al., 2015). It is not known whether this MDH incorporates a lanthanide or calcium is incorporated instead, potentially assisted by the small subunit MxaI. ‘*Ca.* M. lanthanidiphila’ lacked the redundancy in MDHs seen in ‘*Ca.* M. oxyfera’ and encoded for only a single XoxF-type MDH operon (mela_00914-16), with the catalytic subunit showing highest identity to the XoxF2 of ‘*Ca.* M. oxyfera’ (Damo_0134; 83% identity). In ‘*Ca.* M. lanthanidiphila’ the cluster encoding for the calcium-dependent MDH was absent, including the MxaI small subunit. This suggested that ‘*Ca.* M. lanthanidiphila’ utilized a lanthanide-dependent homodimeric XoxF2-type MDH and did not employ the XoxF-MxaI heterodimer as observed in ‘*Ca.* M. oxyfera.’ The genome of ‘*Ca.* M. limnetica’ also possesses only a single copy of the lanthanide-dependent xoxF-type MDH (Graf et al., 2018). As a consequence ‘*Ca.* M. lanthanidiphila’ and ‘*Ca.* M. limnetica’ are most likely dependent on lanthanides to produce an active MDH.

The CSP1-5 genome also encodes for a single MDH (XU15_C00376-379), most similar to the XoxF2 from ‘*Ca.* M. oxyfera’ (Damo_0134; 71% identity). For the coordination of a rare-earth element in the active site of the XoxF-MDH a specific aspartate residue was shown to act as crucial ligand (Pol et al., 2014), which is conserved in all identified XoxF sequences (Keltjens et al., 2014). In both the XoxF of ‘*Ca.* M lanthanidiphila’ and the XoxF of CSP1-5 this aspartate was conserved and was located two positions downstream of the catalytic aspartate. XoxF-MDH is capable of not only oxidizing methanol to formaldehyde, but was also shown to oxidize formaldehyde to formate, thereby circumventing dedicated formaldehyde oxidation systems and preventing the use of formaldehyde for biosynthetic purposes (Pol et al., 2014).

Aside from the abovementioned capability of XoxF-MDHs to oxidize methanol completely to formate, there are two additional formaldehyde oxidation pathways available to ‘*Ca.* M. oxyfera,’ a highly expressed tetrahydromethanopterin (H_4_MPT) (Damo_0454-73) and a less expressed tetrahydrofolate-dependent pathway (H_4_F) (Damo_1852) (Ettwig et al., 2010). ‘*Ca.* M. limnetica’ also possesses both systems. These systems are thought to be active to maintain an optimal balance between catabolism and anabolism of carbon compounds (Chistoserdova, 2011; Reimann et al., 2015). All genes of the two formaldehyde-oxidizing systems were conserved between ‘*Ca.* M. oxyfera’ and ‘*Ca.* M. lanthanidiphila’ (H_4_MPT: mela_2741-2760, H_4_F: mela_00607) as well as in CSP1-5 (H_4_MPT: XU15_C0002G0327-337, H_4_F: XU15_C0002G0238) indicating that this combination of pathways was not only employed by ‘*Ca.* Methylomirabilis’ species, but encompasses more members of the NC10 phylum.

‘*Ca.* M. oxyfera’ has three formyl/formate oxidation systems. The first and highest transcribed system (Damo_0456-60) was part of the tetrahydromethanopterin-dependent formaldehyde oxidation system mentioned above (Reimann et al., 2015). As seen for the whole gene cluster encoding proteins involved in formaldehyde oxidation, this formate/formyl oxidation system was also present in ‘*Ca*. M. limnetica,’ ‘*Ca.* M. lanthanidiphila,’ and CSP1-5 (mela_2743-47, XU15_C0002G0330-34). ‘*Ca.* M. oxyfera’ further encoded two minor expressed NAD(P)^+^-dependent formate dehydrogenases (Damo_1134-1138 and Damo_0853-54). The ‘*Ca.* M. lanthanidiphila’ and ‘*Ca*. M. limnetica’ genomes only encoded for one additional formate dehydrogenase system (mela_1503-04; Graf et al., 2018), which most closely resembled Damo_0853-0854. Similarly, CSP1-5 also harbored one additional formate dehydrogenase (XU15_C0003G0115-16) also most closely resembling Damo_0853-54. Thus all four organisms had the capability to oxidize formate and couple it to the reduction of NAD(P)^+^, thereby providing the cells with extra reducing equivalents.

### Carbon Fixation

The genome of ‘*Ca.* M. oxyfera’ encoded for incomplete C1 assimilation pathways since key genes in the RuMP and serine pathway were absent. Previous genome analysis revealed that ‘*Ca.* M. oxyfera’ harbors all genes of the Calvin–Benson–Bassham (CBB) cycle and activity measurements showed the cycle to be operative (Rasigraf et al., 2014). The ‘*Ca.* M. lanthanidiphila,’ ‘*Ca.* M. limnetica,’ and CSP1-5 genomes contain all genes of the CBB-cycle with genes of the RuMP and serine pathways being absent. The presence of all genes from the CBB pathway for carbon fixation in all four organisms, suggests that the CBB is a conserved feature within the NC10 phylum.

In summary, ‘*Ca.* Methylomirabilis’ species are the only organisms known to couple nitrite reduction to methane oxidation, and the availability of a second and third genome (this article and Graf et al., 2018) added valuable information on the genetic potential and will help to elucidate their basic biochemistry. It appeared that the amendment of micromolar concentrations of the rare-earth metal cerium to the continuous enrichment culture, in combination with the complete removal of nitrate, might have led to a shift from ‘*Ca.* M. oxyfera’ to ‘*Ca.* M. lanthanidiphila.’ Whether ‘*Ca.* M. lanthanidiphila’ was present in the original culture, but not detected in the previous genomic analysis due to low abundance, remains unanswered. Although the inclusion of cerium and the removal of nitrate were the only identifiable differences in the medium between the culturing conditions of the original ‘*Ca.* M. oxyfera’ and the current enrichment it is difficult to link it to the observed shift to ‘*Ca.* M. lanthanidiphila.’ The presence of a lanthanide-dependent XoxF-type MDH as the sole methanol-oxidizing enzyme in ‘*Ca.* M. lanthanidiphila,’ CSP1-5, and in the recently described ‘*Ca.* M. limnetica’ signified the importance of rare earth elements for methylotrophic bacteria of the NC10 phylum and several other phyla (Keltjens et al., 2014).

Based on the conservation of genes for core nitrogen- and carbon-converting pathways between the two ‘*Ca.* Methylomirabilis’ species, and in comparison to the CSP1-5 genome, we observed that CSP1-5 could act as an aerobic methylotroph and has the ability to use nitrate (reduction to nitrite) or sulfate (reduction to sulfite) as alternative electron acceptors (Hug et al., 2016). It appears that the recently discovered intra-aerobic pathway of methane oxidation coupled to nitrite reduction to N_2_ is so far unique to the ‘*Ca.* Methylomirabilis’ genus.

### Description of ‘*Candidatus* Methylomirabilis Lanthanidiphila’

#### Etymology

‘*Candidatus* Methylomirabilis lanthanidiphila’ (lan.tha.ni.di’phi.la. N.L. neut. n. *lanthanidum* lanthanide; N.L. fem. adj. *phila* (from Gr. fem. adj. *phile*) loving; N.L. fem. adj. *lanthanidiphila* loving lanthanides, referring to the need of rare earth elements for an active XoxF-type MDH). The genus name described before (Ettwig et al., 2010) alludes to the substrate methane, which is oxidized by a combination of pathways, involving oxygen as an intermediate.

#### Locality

Enriched from freshwater sediments of a ditch in the Ooijpolder, Netherlands.

#### Properties

Methane-oxidizing and nitrite-reducing bacterium of the candidate division NC10, characterized by phylogenomic analysis of a binned metagenome assembly. The assembled annotated draft genome, which represents the type material, has been deposited under European Nucleotide Archive accession code ERZ663421. Grows anaerobically, but possesses an oxygen-dependent pathway for the oxidation of methane. Reduces nitrite to dinitrogen gas without a nitrous oxide reductase. Gram-negative rod with a diameter of 0.25–0.5 mm and a length of 0.8–1.1 mm. Mesophilic with regard to temperature and pH (enriched at 25–30°C and pH 7–8). Slow growth with an estimated doubling time of 1–2 weeks.

## Data Availability

The unprocessed reads used in this study are available at the European Nucleotide Archive (ENA), under accession code ERS1904634 and the assembled annotated draft genome under accession code ERZ663421.

## Author Contributions

WV, SG-C, MJ, BK, HOC, and JR designed the project and experiments. WV, TA, JF, LG, GC, and DS performed the experimental work. SG-C maintained the cultures. WV, TA, DS, LG, GC, HOC, and JR performed the data analysis and data interpretation. WV, SG-C, BK, HOC, and JR wrote the manuscript with input from DS, JF, LG, GC, TA, and MJ. HC and MJ supervised the research.

## Conflict of Interest Statement

The authors declare that the research was conducted in the absence of any commercial or financial relationships that could be construed as a potential conflict of interest.

## Footnotes

1 https://www.r-project.org

2 http://www.arb-silva.de
