# Supplementary material for: Comparative Genomics of Candidatus Methylomirabilis Species and Description of Ca. Methylomirabilis Lanthanidiphila
Source: Front Microbiol. 2018 Jul 24;9:1672. doi: 10.3389/fmicb.2018.01672 (PMC6094997; doi:10.3389/fmicb.2018.01672)
Supplement: Supplementary file 1 [file Image_1.PDF]

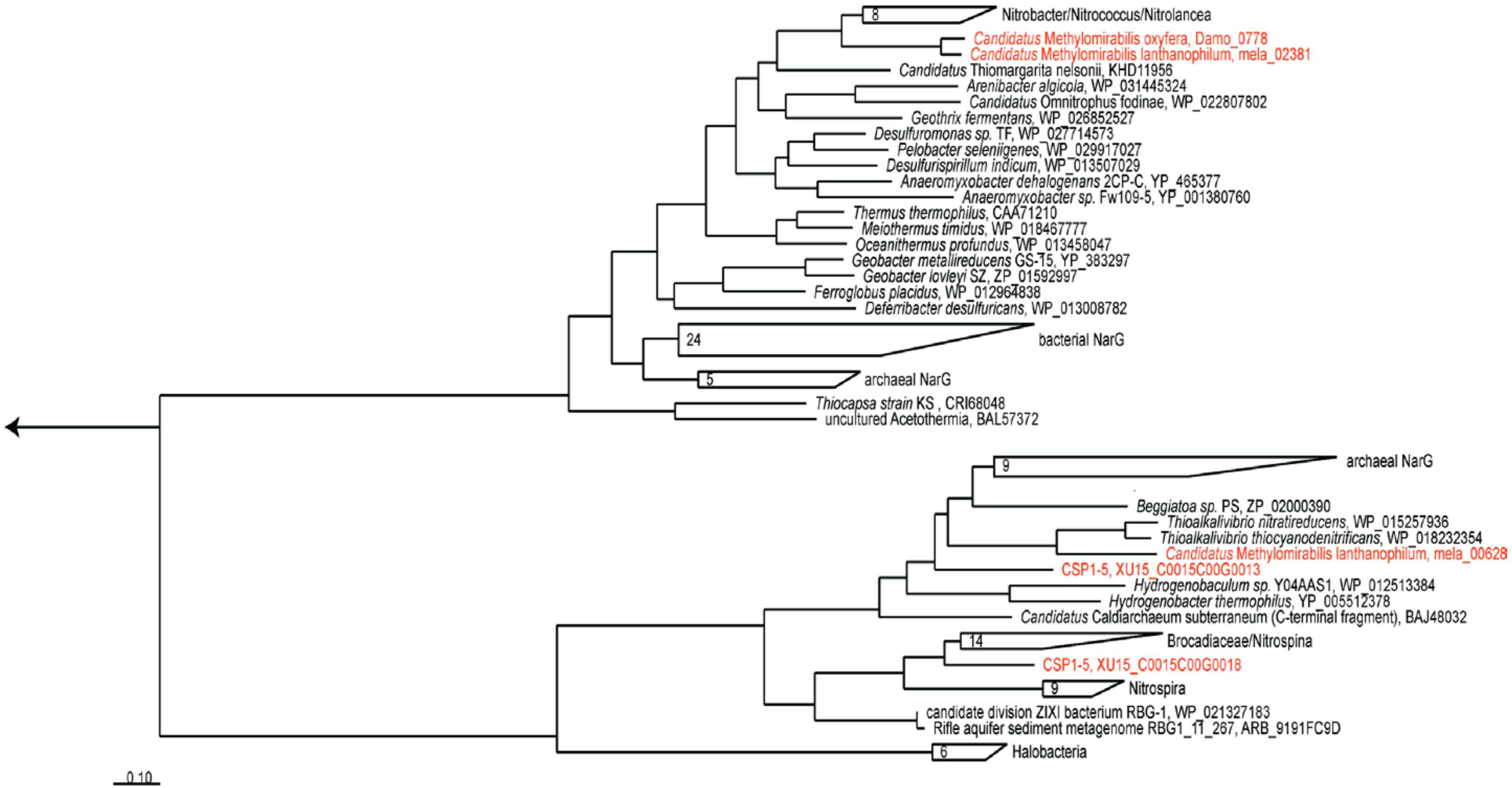

Supplementary FigureS1. Maximum likelihood tree containing representative amino acid sequences of the molybdopterin-binding enzymes of the DMSO reductase type II family. Nar sequences from the NC10 genomes discussed in this paper are indicated in orange. The evolutionary distances were calculated in ARB (version 5.5-org-9167) using PhyML with a 10% conservation filter, which resulted in 1198 validated positions. Numbers indicate the number of sequences within the collapsed node. The outgroup is indicated with an arrow.
